# Expression of concern: The anti-Alzheimer potential of *Tamarindus indica*: an *in vivo* investigation supported by *in vitro* and *in silico* approaches

**DOI:** 10.1039/d4ra90002b

**Published:** 2024-01-11

**Authors:** Abeer H. Elmaidomy, Usama Ramadan Abdelmohsen, Faisal Alsenani, Hanan F. Aly, Shams Gamal Eldin Shams, Eman A. Younis, Kawkab A. Ahmed, Ahmed M. Sayed, Asmaa I. Owis, Naglaa Afifi, Dalia El Amir

**Affiliations:** a Department of Pharmacognosy, Faculty of Pharmacy, Beni-Suef University Beni-Suef 62514 Egypt; b Department of Pharmacognosy, Faculty of Pharmacy, Minia University Minia 61519 Egypt usama.ramadan@mu.edu.eg; c Department of Pharmacognosy, Faculty of Pharmacy, Deraya University, 7 Universities Zone New Minia 61111 Egypt; d Department of Pharmacognosy, Faculty of Pharmacy, Umm Al-Qura University Makkah 21955 Saudi Arabia; e Therapeutic Chemistry Department, National Research Centre (NRC) El-Bouth St., P.O. 12622 Cairo Egypt; f Department of Pathology, Faculty of Veterinary Medicine, Cairo University Giza 12211 Egypt; g Department of Pharmacognosy, Faculty of Pharmacy, Nahda University Beni-Suef 62513 Egypt; h Department of Pharmacognosy, Faculty of Pharmacy, Heliopolis University for Sustainable Development Cairo Egypt

## Abstract

Expression of concern for ‘The anti-Alzheimer potential of *Tamarindus indica*: an *in vivo* investigation supported by *in vitro* and *in silico* approaches’ by Abeer H. Elmaidomy *et al.*, *RSC Adv.*, 2022, **12**, 11769–11785, https://doi.org/10.1039/D2RA01340A.

The Royal Society of Chemistry is publishing this expression of concern in order to alert readers that concerns have been raised regarding the reliability of the photomicrographs in Fig. 2 and 4. An investigation is underway, and an expression of concern will continue to be associated with the article until a final outcome is reached.

Laura Fisher

4th January 2024

Executive Editor, *RSC Advances*

## Supplementary Material

